# Evaluating skeletal muscle wasting and weakness in models of critical illness

**DOI:** 10.1042/CS20255458

**Published:** 2025-07-01

**Authors:** Amy J. Bongetti, Marissa K. Caldow, Yasmine Ali Abdelhamid, Gordon S. Lynch

**Affiliations:** 1 Centre for Muscle Research, Department of Anatomy and Physiology, School of Biomedical Sciences, Faculty of Medicine, Dentistry and Health Sciences, The University of Melbourne, Victoria 3010, Australia; 2 Intensive Care Unit, The Royal Melbourne Hospital, Victoria, Australia; 3 Department of Critical Care, Melbourne Medical School, Faculty of Medicine, Dentistry and Health Sciences, The University of Melbourne, Victoria 3010, Australia

**Keywords:** animal models, cell models, critical illness, inflammation, muscle wasting, muscle weakness, sepsis, skeletal muscle

## Abstract

Skeletal muscle wasting and weakness are common complications associated with admission to the intensive care unit (ICU), with the loss of muscle mass and function increasing mortality and contributing to physical impairments post-discharge. While our understanding of the pathophysiology of this condition, commonly termed ‘ICU-acquired weakness’ (ICU-AW), has advanced considerably, no effective therapies are available. ICU-AW broadly encompasses a range of muscle-related impairments in this setting, including, but not limited to, critical illness myopathy and sepsis-induced myopathy. Pre-clinical models of critical illness can provide insights into the mechanisms underlying muscle wasting and weakness. Cell culture systems can provide mechanistic interrogation, by isolating effects to skeletal muscle directly. Small animal models, like rats and mice, allow for mechanistic investigation of ICU-AW using genetic models and testing pharmacological interventions. Larger animal models, including pigs and sheep, facilitate repeated blood and tissue sampling and can more closely recapitulate the standard-of-care within ICU settings. Although animal models can be advantageous for scientific investigation, they also have important limitations. Barriers to developing effective interventions include difficulty in obtaining muscle biopsies from patients, translating experimental findings between animal models and humans and replicating aspects of different ICU settings. This review explores the advantages and shortcomings of different pre-clinical models of critical illness, identifies gaps in understanding muscle wasting and weakness in critical illness and provides recommendations for improving the translation of therapeutics to promote functional recovery for patients post-discharge.

## Introduction

Up to 50% of patients admitted to the intensive care unit (ICU) experience a loss of muscle mass and function [1], which compromises muscle health and contributes to poor functional recovery post-discharge [2–5]. This phenomenon, first described by William Osler in 1892, as a ‘rapid loss of flesh’ in patients with sepsis [6], is now referred to as ICU-acquired weakness (ICU-AW) [7,8]. ICU-AW has been defined as ‘a syndrome of neuromuscular dysfunction acquired in the absence of causative factors other than the underlying critical illness and its treatment’ [9]. Although understanding of this syndrome has advanced considerably, its aetiology remains to be elucidated.

Patients with ICU-AW can experience an ~20% reduction in quadriceps muscle thickness within 10 days of admission to the ICU [10,11], a decrease of ~2% per day [12]. The extent of weakness is associated with both the severity of critical illness and the degree of systemic inflammation [1]. Patients with ICU-AW can experience chronic disability and reduced quality of life post-discharge [13]. ICU patients can also experience a mortality rate of up to 40% within five years post-discharge [14,15]. Despite the significant impact on health and quality of life, no therapeutics are currently available, with clinical interventions limited to minimising risk factors, such as disuse, and use of neuromuscular blocking agents. An incomplete understanding of the underlying pathophysiology makes ICU-AW difficult to prevent or treat. Appropriate models can provide meaningful insights into the pathophysiology of muscle wasting and weakness associated with critical illness, but barriers exist for clinical translation of these models and the development of effective treatments. To understand these issues, this review will explore the merits and shortcomings of different models of muscle wasting and weakness relevant to critical illness and provide recommendations for improving therapeutic translation. The pathophysiology of ICU-AW will not be discussed in detail since it has been reviewed comprehensively elsewhere [8].

## Clinical diagnosis

The risk of developing ICU-AW increases when patients remain in the ICU for extended periods [16,17]. The current diagnostic method for ICU-AW uses the Medical Research Council (MRC) scale to assess muscle strength. First published in 1943 [18], this method assesses individual muscle strength on a scale of 0–5, with the score determined based on the expected strength of that muscle group [18]. Patients are diagnosed with ICU-AW if they have an MRC sum score of <48 or when the mean score of the 12 muscle groups measured on this scale is less than 4, indicating reduced muscle strength [9,17]. To prevent the progression of ICU-AW and to maintain muscle mass and strength after discharge from the ICU, accurate early-diagnostic methods are required, especially the development of biomarkers that are reliable and minimally invasive.

Other clinical measurements typically utilised in research environments include the assessment of muscle thickness (including the diaphragm), via bedside ultrasound [19,20], and hand grip strength [21]. More invasive measurements include histological assessment of muscles including, but not limited to, the vastus lateralis [11,22,23] and the diaphragm [24]. While specimens obtained via biopsy are highly valuable, collection of a sufficient quantity for conducting relevant assays is not always feasible. Furthermore, muscle samples comprising >150 fibres per sample are required for more accurate cross-sectional area (CSA) analyses [25]. At the whole-body level, functional capacity is typically measured using standardised surrogates such as the six-minute walk test [3] and activities of daily living (ADL) surveys [4]. Integration of these measurements could increase diagnostic capacity, but the requirement for additional labour may not be feasible for all ICUs.

## Components of ICU-AW

### Admission reason, organ failure and inflammation

Critical illness is a key determinant in developing ICU-AW. The severity of illness and inflammation is associated with the degree of impairment and weakness experienced by patients, with septic patients at greater risk of developing ICU-AW [1]. Excessive inflammation impairs muscle health and drives catabolism, contributing to wasting in critically ill patients [26,27]. High levels of systemic inflammation can also contribute to multi-organ dysfunction. The Sequential Organ Failure Assessment (SOFA) score determines the extent of organ failure, which is a well-documented risk factor for developing ICU-AW [11,28]. The SOFA score is specific to critical illness and predicts patient mortality in the ICU according to their level of organ failure [28], with the number of organs in failure being associated with the loss of mass of the rectus femoris muscle [11].

### Disuse

Disuse leads to loss of muscle mass and function [29,30]. Without normal mechanical loading, muscle atrophies due to reduced muscle protein synthesis [31]. In the ICU, this unloading exacerbates the functional decline in patients [32] but is not the sole driver of muscle dysfunction. In a prospective study, after two weeks of immobilisation, patients with sepsis (*n* = 13) and age-matched healthy controls (*n* = 7; lower arm casting) experienced reduced muscle strength of the adductor pollicis muscle after a fatiguing protocol of supramaximal ulnar nerve stimulation [33]. However, unlike immobilised healthy controls, patients with sepsis also experienced reduced maximum strength with supramaximal ulnar nerve stimulation, highlighting the additional complexity and impact on muscle function associated with critical illness, compared with immobilisation alone.

Early mobilisation in ICU patients can be highly effective in attenuating weakness at discharge [34,35]. Exercise-based interventions are also effective in association with standard care. A 20-minute session of either passive or active leg cycling five days per week increased functional capacity (based on a six-minute walk test) and isometric quadriceps strength after hospital discharge [36]. However, cycle ergometry is not a feasible option for all patients. While current guidelines outline safety criteria for the mobilisation of patients, even for those that are mechanically ventilated [37], this is not always possible due to the severity of illness and because of staffing limitations [38,39]. Passive interventions like electrical muscle stimulation (EMS) to mimic exercise do not require mobilisation [40], but efficacy remains unclear based on the conflicting evidence presented in systematic reviews [41], and the variability in stimulation characteristics, including pulse and EMS duration [42].

### Mechanical ventilation

Many patients admitted to the ICU are mechanically ventilated [43,44]. Prolonged mechanical ventilation can cause diaphragm weakness due to unloading of this tonically contracting muscle [45], causing delays for patients to be weaned from mechanical ventilation [46–48], which compromises health and increases mortality [44,49,50]. Mechanical ventilation is also independently associated with ICU-AW [51–56] and with an increased mortality in these patients [57].

Modification of patient care to address some of these factors has improved patient outcomes after discharge from the ICU. Neuromuscular blockers (NMBs) facilitate intubation in patients which can optimise mechanical ventilation [58], but since NMBs are competitive acetylcholine antagonists that bind to nicotinic receptors on the motor endplate, they prevent excitation of the muscle [59]. This can potentially exacerbate the effects of disuse, which is a known risk factor for ICU-AW. Use of these agents in the clinic has declined during the 21st century due to improvements in clinical practice and ventilatory support, thus limiting patient risk [58,60]. Isolating the direct effects of disuse/immobilisation from sedation, mechanical ventilation and NMBs, is difficult, but disuse in the absence of critical illness can cause significant muscle atrophy [29,30].

## Risk factors

The diagnosis and pathophysiology of ICU-AW is made more complex by different risk factors, including age [61], sex [52] and nutrition status [62]. Heterogeneity within the patient population serves as a considerable barrier for elucidating the pathophysiology of ICU-AW and for the design of meaningful clinical studies. To overcome these barriers, it is imperative to understand the populations most at risk, for better patient stratification and for developing and refining pre-clinical models.

While current understanding of the risk factors that lead to muscle wasting and weakness relate to the typical ICU population, which is often patients of a more advanced age, further investigation is needed of the muscle wasting and weakness in younger patients (infants, children and young adults). Furthermore, accounting for differences between biological sex as a risk factor within these categories is essential for developing a more personalised standard of care for ICU patients to optimise their recovery.

### Age

Sarcopenia, the loss of muscle mass with advancing age, contributes to frailty and the large burden of disease in the elderly [63]. A large proportion of patients admitted to the ICU are of advanced age and more likely to present with compromised muscle health, including sarcopenia, that contributes to adverse functional outcomes post-discharge [61].

While advanced age considerably affects susceptibility to ICU-AW, younger adults [64] and paediatric [65] patients still experience muscle wasting and weakness after ICU admission. Younger adult and paediatric patients are not typically included in longitudinal follow-up studies which may bias mechanistic investigations. Similarly, further clinical investigation into potential differences in the mechanisms of ICU-AW in paediatric, adult and aged populations is required to provide more targeted treatments and standard of care.

### Sex

Women admitted to the ICU have significantly higher mortality rates [66], are at greater risk of developing ICU-AW [52,67,68] and experience greater reductions in the CSA of type IIA fibres in the vastus lateralis muscle compared with men [69]. Analysis of serum hormonal status in 37 post-menopausal women with ICU-AW revealed no hormonal dysfunction compared with patients without ICU-AW [68]. Despite these significant findings, population bias in these studies confounds the accuracy of their interpretation to the female sex. It is currently unknown whether women with a regular menstrual cycle are equally susceptible to ICU-AW as post-menopausal women. While studies in animal models of critical illness are limited, female mice exhibited a different wasting profile in response to disuse atrophy compared with male mice [70]. Female mice had an earlier onset of wasting in the tibialis anterior (TA) muscle, and greater overall reductions in type IIB fibre CSA compared with male mice over 168 hours of disuse [70]. As there were no experimental modulations of ovarian function (i.e. ovariectomy), it is unclear whether the changes were mediated by ovarian hormones. Further studies, without the confounding factor of age, i.e., in amenorrheic women under 50, are required to confirm and uncover the mechanisms underlying these reported sex-specific differences.

### Malnutrition

Nutrition plays an important role in protein synthesis and is essential for muscle development, for preserving muscle mass and for promoting muscle regeneration [71]. Inadequate intake of protein and amino acids can exacerbate muscle atrophy, especially in patients with cancer cachexia [72], sarcopenia [73] and ICU-AW [62]. Patients are more likely to retain muscle mass with adequate nutrition in the first seven days of admission to the ICU compared with patients who do not achieve energy requirements [74]. Malnourished patients in the ICU had significantly decreased CSA of the pectoralis major muscle measured by CT scan compared with non-malnourished counterparts [75]. However, despite adequate feeding, critically ill patients can also have a blunted capacity for protein synthesis, termed ‘anabolic resistance’ [76]. Incorporation of phenylalanine labelled protein into the muscle was 60% lower in ICU patients compared with healthy controls, despite matched amino acid availability in the systemic circulation and similar rates of fasting myofibrillar protein synthesis [76]. Although adequate nutrition may not necessarily reverse the loss of muscle mass, it may help attenuate the severity of wasting and possible complications after hospital discharge.

## Skeletal muscle wasting and weakness in critically ill children

Unlike the adult ICU population, the incidence of ICU-AW in the paediatric ICU (PICU; <18 years) is considerably lower, with reported cases between 0.02% and 1.7% [77,78]. Ultrasound measurements from 30 PICU patients revealed ≥10% loss of muscle mass of the lower limb and diaphragm occurring within five to seven days of admission [79]. Limited data for lower limb biopsies in three patients revealed ‘scattered basophilic atrophic fibres with loss of ATPase reactivity’ associated with loss of myosin and increased calpain activity, consistent with a diagnosis of acute quadriplegic myopathy [77]. Persistent weakness or altered endurance capacity was experienced by survivors 3–18 months after hospital discharge [77].

Within the PICU, older patients (more than ten years) were significantly more likely to develop ICU-AW, particularly after onset of puberty (5.1% incidence) compared with infants (three months to three years; 0.7% incidence) [77]. Older children (more than one year of age) were more likely to experience wasting of the lower limb compared with infants (less than one year old) [79].

There is currently an insufficient understanding of the pathophysiology, risk factors and long-term consequences on muscle function and development after critical illness in children [80]. The complexity in paediatric ICU-AW highlights the importance of further investigations into mechanisms associated with age and the onset of puberty, as well as long-term functional outcomes, regardless of age.

## Skeletal muscle wasting and weakness in patients after ICU discharge

The mechanisms underlying inadequate recovery from ICU-AW remain poorly understood. Patients with ICU-AW are more susceptible to long-term skeletal muscle dysfunction after discharge [5,81], with physical impairments persisting for up to five [3] or ten years [82]. In a prospective, longitudinal cohort study, physical dysfunction was evident for up to five years after acute respiratory distress syndrome (ARDS), with patients only able to achieve 76% of their predicted functional capacity in the six-minute walk test [3]. The impaired recovery was despite an initial improvement at one year, after which patients reached a plateau in the restoration of muscle function. Survivors of severe sepsis also experienced long-term functional impairments based on their performance of ADL and instrumental ADLs surveys [4]. Long-term abnormalities (6–24 months) in motor and sensory nerve conduction were not attributed to a specific sub-population (based on age, gender, diagnosis of sepsis or time after ICU discharge) [83]. Despite reports of significant long-term dysfunction, more comprehensive longitudinal studies are required to better understand the trajectory of wasting and weakness in patients across different ages, sex and critical illness diagnoses.

Persistent muscle weakness and fatigue with ICU-AW are attributed to a poorly understood, heterogeneous skeletal muscle pathology [2,84]. Few clinical studies have investigated the mechanisms of long-term wasting and weakness. Six months after discharge, most patients showed improvements in muscle CSA measured via CT scan, but these were still lower than in age- and sex-matched controls [2]. Quadriceps muscle CSA did not correlate with muscle strength, based on isometric peak torque measurements [2]. The same study examining vastus lateralis muscle samples revealed that patients who did not exhibit a restoration of muscle mass had reduced satellite cell (muscle stem cell) content, possibly indicative of impaired muscle regenerative capacity and poor muscle health [2]. Comorbidities may also be a significant risk factor for some, but not all patients experiencing long-term dysfunction [2]. Extrapolation of these results from these studies is limited due to the small sample size (11–15 patients). Furthermore, very few studies investigate the efficacy of tailored intervention programmes to enhance recovery using adequate control groups [85]. Most longitudinal follow-up studies investigate patients during mid-life or older, with few studies including younger adults and children. Further mechanistic investigation into long-term functional outcomes is required.

## Barriers to understanding the mechanisms underlying ICU-AW for therapeutic development

ICU-AW and long-term functional impairments carry a high burden of disease and severely affect the quality of life for patients [86]. Despite the magnitude of these long-term deficits, few studies have evaluated the mechanisms responsible for persistent muscle dysfunction. This dearth of knowledge is attributed to the high rates of attrition post-hospital discharge [87] and because most studies focus on the initial insult of disease, such as sepsis, rather than the long-term consequences after the life-threatening critical illness has resolved. Further investigation into risk factors and mechanisms relating to age and sex stratification is needed to better inform patient care.

Despite a reasonable understanding of the various causative factors for ICU-AW, treatment efficacy is limited. Although a greater mechanistic understanding of the syndrome could be achieved from muscle biopsies obtained from patients, such studies are limited by logistical challenges, including the need for sufficient sample sizes for statistical power and meaningful physiological analyses. The collection of diaphragm muscle biopsies, although desirable for such mechanistic investigations, is obviously technically challenging because of its anatomical location and function.

There are clear gaps in our understanding of critical illness and ICU-AW that could be addressed through the development, optimisation and use of cell and animal models. These include [1] identifying the mechanisms underlying muscle wasting and weakness in critical illness relevant to ICU-AW; [2] identifying effective nutritional, pharmacological and other interventions (including exercise and electrical stimulation) that can counter muscle loss and physical dysfunction associated with critical illness; [3] identifying whether some muscle groups are more susceptible to weakness and wasting in critical illness, including comparisons between limb muscles and the diaphragm; and [4] identifying whether there are age and sex-specific differences with respect to the susceptibility to muscle dysfunction during critical illness and recovery post-discharge from the ICU. To understand the origins of these gaps in knowledge and to address these barriers to improve treatment efficacy, rigorous pre-clinical investigations in critical illness settings are important for evaluating therapeutic potential. Cell and animal models may provide greater mechanistic insights applicable to specific clinical settings, mindful of their obvious limitations. This review describes the advantages and limitations of some of the models employed in pre-clinical studies of critical illness relevant to ICU-AW and provides suggestions for improving clinical translation.

## Skeletal muscle wasting and weakness in animal models of critical illness

**Figure 1:**
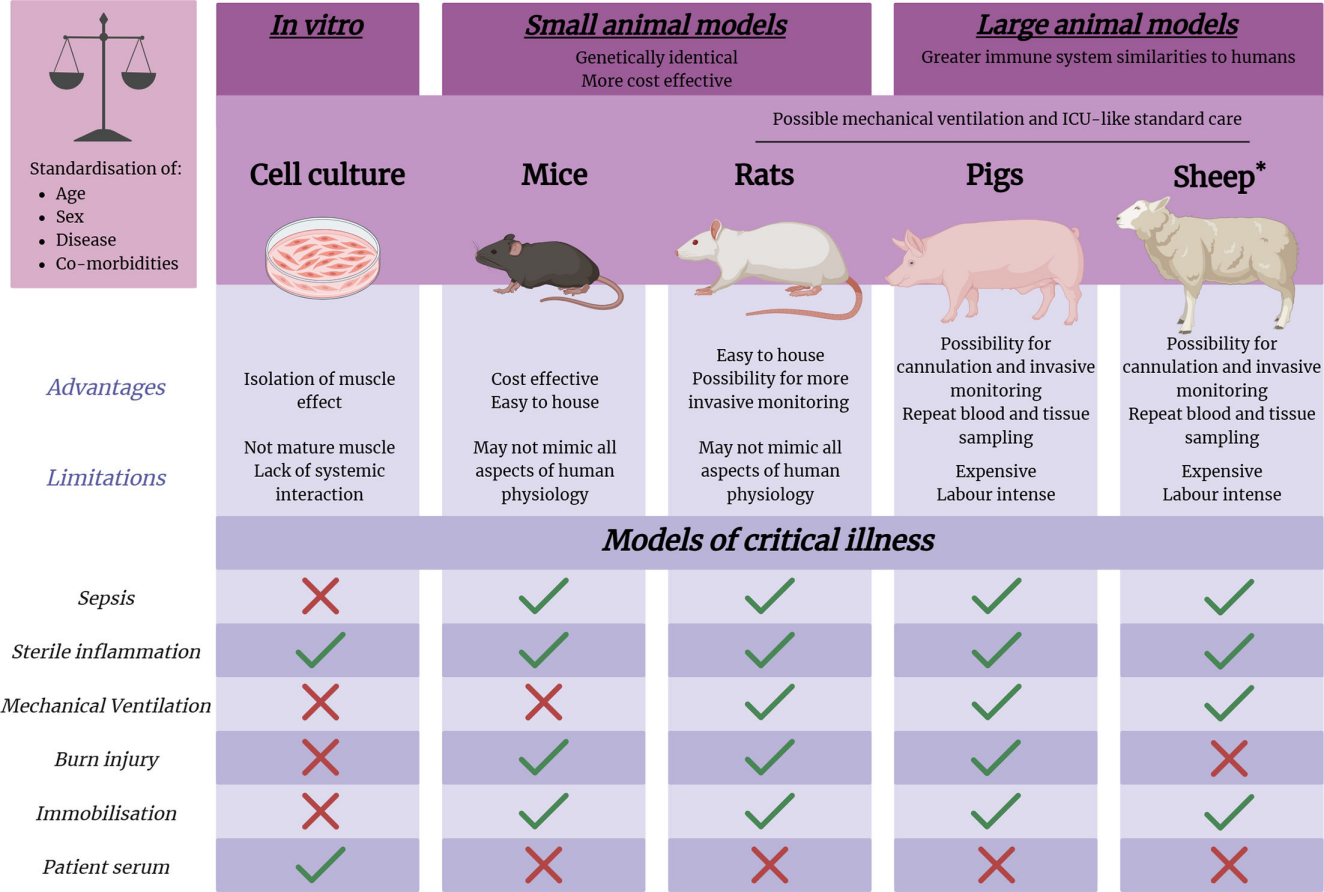
Strengths and limitations of different models of muscle wasting and weakness relevant to critical illness. Muscle wasting and weakness with critical illness and intensive care unit (ICU) admission can be highly debilitating for patients, with significant impact on quality of life. Currently, there are no approved clinical treatments. Models of critical illness, including cell culture systems, and small and large animal models, provide experimental platforms for mechanistic interrogation and understanding how risk factors, such as age, sex and co-morbidities, contribute to an acquired myopathy. *In vitro* systems help isolate the effects of critical illness on skeletal muscle directly, providing excellent high-throughput screening of potential therapeutic candidates, despite lacking an integrated physiological system like in intact animal models. Small animals such as mice and rats are relatively cost-effective experimental platforms compared with larger animal models such as pigs and sheep, and their genome can be edited more easily for identifying specific genes in the conferral of protection from and/or susceptibility to wasting and weakness with critical illness. In contrast, larger animals have an immune system like that in humans with the capacity for more invasive monitoring and repeated blood and tissue sampling. *Although sheep are utilised for sepsis research, they are not commonly utilised for studying muscle wasting and weakness in critical illness. Ticks represent the disease phenotype induced in each of the different models, and crosses indicate these models are not commonly utilised for studying these conditions. Created in BioRender. Bongetti, A. (2025) https://BioRender.com/.

Pre-clinical animal models of critical illness can control for risk factors such as age, sex and disease co-morbidities, reducing genetic variability and increasing statistical power. Compared with *in vitro* cell culture models (described in detail later), experimental animals facilitate investigation of intact physiological systems and their interactions. Small animals, like rats and mice, allow for greater mechanistic investigation of specific aspects of critical illness relevant to ICU-AW through genetic and pharmacological manipulations. Larger animals, including pigs and sheep [8,88], allow for repeated blood and tissue sampling and can more closely recapitulate the standard-of-care in ICU settings. Animal models also allow for examination of different muscle groups with different muscle fibre type composition, which may be relevant when describing treatment outcomes and potential clinical relevance. The strengths and limitations of the different models of muscle wasting associated with critical illness are summarised in Figure 1 [88,89].

### Models of sepsis

Sepsis is a leading cause of ICU admission and mortality worldwide [90] and an independent risk factor for developing ICU-AW [1]. Many inflammatory models can lead to multi-organ dysfunction and/or failure [91,92], which are predictive factors for ICU-AW [11,52,86]. Various models of sepsis have provided important information about the time course of sepsis development [93] and are key to investigating the mechanisms responsible for muscle wasting and weakness in patients with sepsis.

#### a) Caecal ligation and puncture

The caecal ligation and puncture (CLP) procedure, a model of polymicrobial sepsis, involves the isolation, ligation (distal to the ileocaecal valve) and puncture of the caecum via laparotomy [94–96]. The severity of the CLP model depends on the length of the ligation, size and number of punctures, and whether antibiotics are administered [94–96]. CLP is often described as the ‘gold standard’ of small animal (rodent) models of sepsis, as it closely resembles the progression of sepsis in humans [96]. Administration of antibiotics, analgesics and fluid resuscitation in these models can resemble the standard of care received by patients in the ICU.

In rodents, the CLP model can lead to significant reductions in body mass, muscle mass and muscle function [97–100]; a skeletal muscle phenotype often exhibited by critically ill patients. Generally, muscle mass loss is evident as early as 24 hours post CLP surgery in mice [101] and at 96 hours [100], 5 days [102,103] and 7 days [98]. Variations in model severity mean that comparisons between studies are not always seamless, with differences in mortality rates and severity of sepsis, endpoint and muscle groups analysed. Even considering these differences, the CLP model is highly informative, especially when making comparisons between studies. As with sepsis in patients, increased muscle protein catabolism and reduced protein synthesis have also been observed in mice, rats and pigs using this model [101,104–106]. Although the CLP model in pigs is highly effective and offers considerable advantages as a model system, including its greater immune system capacity and provision for invasive monitoring and repeat blood collection, rodents are utilised more commonly because they are more cost effective and require less direct researcher support.

In patients, sepsis-induced myopathy is associated with reduced muscle function. In septic mice, five days post CLP surgery, electromyography (EMG) of the sciatic nerve-gastrocnemius muscle complex revealed a significantly increased amplitude, with signs of axonal swelling compared with controls [99]. However, both indirect nerve stimulation and direct muscle stimulation of the extensor digitorum longus (EDL) muscle revealed impaired maximum absolute force generating capacity in septic mice, indicating that effects intrinsic to the muscle were responsible for the dysfunction rather than neural abnormalities [99]. Animals in this study were six months of age, older than those typically used in sepsis research (10–12 weeks), which increases translational relevance to adult populations. However, these studies would benefit from examining differences in aged mice (>18 months old) to examine how sepsis affects muscle health in the elderly, especially with respect to neuromuscular dysfunction. Experiments on isolated single EDL muscle fibres to interrogate mechanisms pertaining to reduced functional capacity showed that contractile dysfunction in septic mice was not attributed to aberrant Ca^2+^-handling but to sarcomere disorganisation, with muscle fibres being more susceptible to damage even when subjected to a protocol of passive stretching [99]. Using rodents in sepsis research allows for the assessment of muscle function either *in situ* with nerve and blood supply intact or *ex vivo* on isolated muscles, facilitating investigation of neuromuscular mechanisms in ways that cannot be replicated in patients. Septic rats exhibited altered sodium channel properties, 10 days after CLP surgery [107], and fibres isolated from the flexor digitorum brevis muscle had reduced maximum sodium current and sodium conductance, indicative of muscle inexcitability [107].

Time course analysis of wasting in the tibialis anterior muscles of mice after CLP surgery revealed an initial increase in local inflammatory signalling and ubiquitin-dependent catabolic processes 24 hours and 96 hours post sepsis induction [100]. A more comprehensive analysis of wasting (12, 24, 48 and 72 hours) after CLP identified three specific transcriptional phases: inflammatory-immune (0–12 hours), inflammatory-atrophy (24 hours) and atrophy (48–72 hours) [108], revealing important insights into the initiation of muscle wasting with sepsis. Given that analyses were limited to one muscle group [108], it is possible that different muscles with different fibre type compositions may be affected differently by sepsis. Identifying potential muscle/fibre type specific responses to sepsis could help inform therapeutic development.

Genetic manipulations in mouse models provide advantages for interrogating the mechanisms of disease. Muscle-specific deletion of *Il6st* revealed the importance of gp130/JAK2/STAT3 signalling in mediating muscle wasting after CLP surgery, with a partial attenuation of wasting in the tibialis anterior and gastrocnemius/plantaris muscle complex compared with wildtype (WT) CLP counterparts 96 hours post-surgery [100]. Genetic ablation of muscle stem cells (satellite cells) impaired recovery of muscle fibre size in tibialis anterior muscles 30 days after CLP surgery [109], which coincided with a blunted capacity for CLP mice to recover from a secondary muscle injury (induced by intramuscular injection of barium chloride) even four weeks post damage [109]. These studies investigating the role of muscle stem cells in recovery from sepsis-induced muscle wasting are important for understanding why many older patients in the ICU experience a decline in muscle health, attributed potentially to an age-related impairment in regenerative capacity [110]. Studies using older (rather than young or adult) mice age would likely yield greater mechanistic insight into the muscle wasting and weakness in critically ill older adults, who make up a large proportion of the ICU patient population.

The CLP model has been described as the ‘gold standard’ small animal (rodent) model of sepsis because it closely resembles the progression of sepsis in humans [96] and has been very well characterised. However, despite the widespread use of the CLP model, it is not a model of severe, chronic sepsis and is not typically suitable for long-term studies because it is associated with a high mortality. Studies incorporating antibiotics and fluid resuscitation interventions with the CLP model report considerably lower mortality rates, with animals surviving for up to 50 days after CLP surgery [109]. Differences in the severity and duration of the various models need to be considered when interpreting different study findings. Another important consideration for this model is that the consequences from CLP have their origins in the peritoneal cavity, which, although clinically relevant, since ~21% of septic patients present to the ICU with some form of peritonitis [111], the aetiology differs from the majority of patients.

#### b) Administration of live bacteria

A common method of inducing sepsis in small [112] and large animal models [113,114] is the administration of an intraperitoneal (*i.p*) or intravenous (*i.v*) bolus or infusion of bacteria, either, in mice [115,116], rats [117], pigs [118,119], sheep [113,120], dogs [121] and non-human primates, such as baboons [122]. Rodents and pigs are the most employed models for studying the relationship between sepsis and skeletal muscle wasting and weakness [112,123,124]. Muscle atrophy has been observed 7 and 14 days after administration of *Escherichia coli* to rats [112], which was associated with reduced maximum tetanic force (strength) of the tibialis cranialis muscle *in situ,* not attributed to a change in the number of acetylcholine receptors [112]. Although this suggests a potential intrinsic deficit in muscle function, closer interrogation of the neuromuscular dysfunction would be required to be definitive.

Studies utilising administration of *E. coli* are typically of longer duration than CLP models, which are usually terminal seven days after induction of sepsis. A longer time course resulting in muscle wasting for investigation of prolonged deficits in muscle mass and function, like those in many patients who are in the ICU for extended periods. Furthermore, *i.v*. administration of *E. coli* allows for investigation of muscle wasting and weakness associated with sepsis originating within the systemic circulation, which is more clinically relevant than many other sepsis models where the effects of critical illness originate in the peritoneal cavity.

Intranasal delivery of *Streptococcus pneumoniae* [125] provides an alternative induction route of sepsis, utilising the less common gram-positive sepsis with origins in the lungs, an induction method highly relevant to patients that present with respiratory-derived sepsis (~70% of septic patients) [111]. Delivery via this method in mice induced diaphragm muscle weakness after five days, evident from the decrease in maximum force production of isolated diaphragm muscle strips *ex vivo* [125]. There was no change in forelimb grip strength over the study duration, but assessments of muscle size or hindlimb muscle function were not conducted. Further investigations of muscle structure–function are needed to properly assess its merits as a model of critical illness and relevance to ICU-AW.

Although bacterial administration is a reliable method for investigating the host response to infectious mediators, bacterial strains do not typically replicate *in vivo*. Large doses of bacteria are typically required to overcome host defence mechanisms [126] and initiate an aberrant inflammatory response. Furthermore, studies in animal models commonly require a continual infusion of bacteria, rather than a bolus administration, which does not recapitulate the induction of sepsis in patients [112], and limits their clinical translation.

#### c) Faecal (caecal) slurry

Sepsis can also be induced through the harvesting of material found in the large intestine, termed a faecal or caecal slurry (CS). In these cases, the contents of the bowel are combined with saline and other reagents, such as glycerol, to create a suspension or slurry of known concentration, which is then injected (*i.p*.) into either mice [127], rats [128] or pigs [129]. Atrophy of the tibialis anterior muscles and reduced forelimb grip strength have been reported in mice up to 61 days post-injection [130], with wasting attributed to increased neutrophil infiltrate 14 days after CS injection. Neutrophil-specific depletion using Ly6G antibody increased forelimb grip strength and muscle CSA compared with CS injection alone, confirming the essential role of neutrophils in muscle wasting with critical illness. Unlike other sepsis models in mice, the survivability of mice for up to three months after sepsis induction allows for investigation of a muscle wasting phenotype like that in patients hospitalised for extended periods. This model has also been utilised in other model organisms, with nitric oxide overproduction and mitochondrial dysfunction (complex I inhibition and ATP depletion) evident in skeletal muscles of rats [131]. Electrophysiological abnormalities have also been reported in this model, with reports of membrane depolarisation and sodium channel inactivation in the muscles of pigs [132].

Studies in rats and pigs have yet to associate the induction of sepsis with muscle wasting and weakness in the CS model, and mice remain the preferred species for assessment due to the comprehensive testing that can be undertaken. Studies in mice are also advantageous as they permit investigations relevant to long-term dysfunction after ICU discharge. One month after CS injection, mice experienced a loss of intrinsic or specific force (maximum strength normalised to muscle cross-sectional area), in isolated EDL muscles *ex vivo,* without the loss of muscle mass [133]. Ankle plantar flexion peak torque (assessed *in vivo*) remained compromised for up to 70 days after sepsis induction [134], despite resolution of systemic inflammation. The impaired muscle strength was attributed to a reduced abundance of mitochondrial respiratory chain complexes, I, II and IV [133].

Although proposed as an alternative to CLP because of its less invasive methodology, limitations of the CS model include the inability to standardise microbial type and concentration, making its severity and mortality outcomes difficult to reproduce. Attempts to refine the variability in the model have been proposed elsewhere [135].

Despite the restoration of muscle mass, the CS model is one of the first models of long-term dysfunction [133,134] and the capacity to interrogate mechanisms underlying muscle wasting and weakness in the long term after critical illness is particularly advantageous. The long-term CS model also offers potential for testing novel therapeutics in settings relevant to patients in the ICU.

### Other models of sepsis not used for studying muscle wasting and dysfunction

Other animal models for studying the progression of sepsis and associated treatments include colon ascendens stent peritonitis (CASP), implantation and abscess models, although these are not typically employed for studying sepsis-induced muscle wasting and weakness. Investigations of the skeletal muscle phenotype in these models would help determine their suitability for studying muscle dysfunction in settings of critical illness. The CASP model was developed to standardise sepsis in animal systems [136]. In mice or rats, it involves a similar midline laparotomy like in the CLP model, followed by insertion of a stent into the ascending colon, allowing faecal contents to flow into the peritoneum, resulting in sepsis [137]. Mortality is controlled through regulation of stent diameter. Unlike the CLP procedure, CASP involves a constant leakage of bowel contents into the peritoneal cavity, causing ongoing infection, compared with intra-abdominal abscess formation in the CLP model [126].

While most models of sepsis are either polymicrobial or utilise gram-negative bacteria, the fibrin clot implantation model allows for investigation of gram-positive sepsis. The fibrin clot is impregnated with *Staphylococcus aureus* and placed into the peritoneal cavity in mice after a small laparotomy [138]. Similar models involving implantation include wicks containing faecal material embedded into the thigh muscle in dogs [139,140], and formation of a dermal abscess in rats after implantation of *Pseudomonas aeruginosa* underneath a skin graft [141,142]. *Staphylococcus aureus* can also be placed into the lower lobe of the lung to induce sepsis [143]. This model, combined with mechanical ventilation, is used for studying sepsis with origins in the lungs (pneumonia), which is a common site of infection in patients. It is unclear whether these models exhibit a muscle wasting phenotype, but they could provide insight into muscle pathologies associated with septic insults from various origins.

### Models of sterile inflammation and endotoxemia

Since high levels of inflammation in critically ill patients are associated with ICU-AW, different models of inflammation are used to study the pathophysiology of ICU-AW (Figure 1) [26,27]. Sterile inflammation, induced by pathogen-associated molecular patterns (PAMPs) such as lipopolysaccharide (LPS) or zymosan, activates Toll-like receptors (TLRs) and can mimic the systemic inflammation associated with sepsis [144]. Typically administered *i.p*. or *i.v*. to model organisms, these serve as good platforms for studying the acute inflammatory response associated with infection (Table 1) [126]. Isolation of the toxin allows for standardisation of the inflammatory response, which is more difficult with polymicrobial and live-inoculation bacterial models. Furthermore, the sterile nature of the PAMP allows for their use in cell culture systems to mechanistically interrogate the inflammatory response and the cellular responses to therapeutic interventions.

**Table 1:** Models of muscle wasting and weakness associated with critical illness.

Animal/ Cell type	Pathology induced (administration route)	Welfare support	Skeletal muscle phenotype	Clinical phenotype and translational weaknesses		Ref.
				Strengths	Weaknesses	
**Caecal ligation and puncture (CLP**)						
Mice	Sepsis; polymicrobial (surgical)	Fluid resuscitation, antibiotics and analgesic optional	Muscle atrophy and dysfunction Mitochondrial dysfunction Increased protein catabolism Reduced protein synthesis Disorganised sarcomeres Anabolic resistance	Sepsis progression similar to humans ICU-like standard care Loss of mass and dysfunction Inflammatory response Altered protein balance	Usually short duration No mechanical ventilation or immobilisation	[98–101,145,146]
Rats	Sepsis; poly-microbial (surgical)	Fluid resuscitation and analgesic optional	Muscle atrophy and dysfunction Mitochondrial dysfunction Increased protein catabolism Reduced protein synthesis Disorganised sarcomeres Hypoexcitability	Sepsis progression similar to humans ICU-like standard care Loss of mass and dysfunction Inflammatory response Altered protein balance Nerve pathology	Usually short duration No mechanical ventilation or immobilisation	[104,105,107]
Pigs	Sepsis; polymicrobial (surgical)	Fluid resuscitation, haemodynamic maintenance, analgesic and anti-inflammatories	Muscle atrophy Increased protein catabolism Reduced protein synthesis	ICU-like standard of care Loss of mass Altered protein balance Repeat blood sampling Immune system similarity to humans	No mechanical ventilation or immobilisation Cost prohibitive Labour intense	[106]
**Inoculation with live bacteria**						
Mice	Sepsis; *Escherichia coli* (*i.p*.)	Limited	Muscle atrophy	Loss of mass	Usually short duration No mechanical ventilation or immobilisation	[147]
Rats	Sepsis; *Escherichia coli* (*i.p., i.v*.)	Limited	Muscle atrophy and dysfunction	Loss of mass and dysfunction Inflammatory response	No mechanical ventilation or immobilisation	[112,123,148]
Mice	Sepsis; *Streptococcus pneumoniae* (intranasal)	Fluid resuscitation with antibiotics	Reduced contractility of the diaphragm	ICU-like standard care Inflammatory response Muscle weakness of the diaphragm	Short duration No hindlimb wasting or weakness No mechanical ventilation or immobilisation	[125]
Pigs	Sepsis; *Pseudomonas aeruginosa* (*i.v*.)	Fluid resuscitation	Muscle inflammatory infiltrate	ICU-like standard of care Repeat blood sampling Immune system similarity to humans	No muscle wasting or dysfunction No mechanical ventilation or immobilisation Cost-prohibitive Labour intense	[149]
**Faecal (caecal) slurry**						
Mice	Sepsis; polymicrobial (*i.p*.)	Limited	Muscle atrophy and dysfunction Inflammatory infiltrate	Loss of mass and dysfunction Inflammatory response Capacity for investigations into long-term dysfunction	No mechanical ventilation or immobilisation Standardisation can be difficult	[130,133,134]
Rats	Sepsis; polymicrobial (i.p.)	Fluid resuscitation	Biogenetic abnormalities	ICU-like standard care Mitochondrial dysfunction	No mechanical ventilation or immobilisation Standardisation can be difficult	[131]
Pigs	Sepsis; polymicrobial (surgical)	Fluid resuscitation, antibiotics	Electrophysiological abnormalities	ICU-like standard care Mechanical ventilation and immobilisation	Cost-prohibitive Labour intense	[132]
**Sterile inflammation and endotoxemia**						
C2C12 cells	LPS; Systemic inflammation	N/A	Myotube atrophy Reduced fusion index Inflammation Increased catabolism Increased autophagy	Examination of muscle in isolation Atrophy Increased catabolism	Absence of systemic interactions	[150–152]
Mice	LPS; Systemic inflammation (*i.p., i.v., intratracheal*)	Limited	Muscle atrophy and dysfunction Increased inflammation Increased catabolism Reduced protein synthesis Increased autophagy Anabolic resistance	Loss of mass and dysfunction Inflammatory response Altered protein balance Anabolic resistance	Acute inflammatory response Usually short in duration No mechanical ventilation or immobilisation	[150,151,153–156]
Rats	LPS; Systemic inflammation (*i.p., i.v*.)	Fluid resuscitation	Muscle atrophy and dysfunction Increased catabolism Reduced oxidative capacity	Loss of mass and dysfunction Inflammatory response Altered protein balance	Acute inflammatory response Usually short in duration No mechanical ventilation or immobilisation	[157–161]
Guinea pig	LPS; Systemic inflammation (Intranasal)	Limited	Muscle atrophy	Loss of muscle mass	No mechanical ventilation/ immobilisation	[162]
Pigs	LPS; Systemic inflammation (*i.p, i.v*.)	Fluid resuscitation, haemodynamic maintenance	Muscle atrophy and dysfunction Increased catabolism Reduced protein synthesis pathways	ICU-like standard care, Loss of mass and dysfunction Inflammatory response Altered protein balance Immune system similarity to humans Mechanical ventilation and immobilisation	Cost-prohibitive Labour intense	[163–166]
Rats	Zymosan; Peritonitis (*i.p*.)	Limited	Muscle atrophy and dysfunction Increased catabolism Reduced protein synthesis Increased autophagy	Loss of mass and dysfunction Inflammatory response Altered protein balance Recovery model	No mechanical ventilation or immobilisation	[167,168]
**Corticosteroids with denervation**						
Rats	Sciatic nerve denervation, corticosteroids	N/A	Muscle atrophy and dysfunction Disorganised sarcomeres Electrophysiological abnormalities	Loss of mass and dysfunction Altered protein balance	Nerve primarily intact in patients (trophic signalling from endplate) No critical illness No mechanical ventilation or immobilisation	[169–171]
**Intensive Care Unit (ICU**)						
Rats	Immobilisation, mechanical ventilation, neuromuscular blockers	Limited	Muscle atrophy and dysfunction Increased catabolism Reduced protein synthesis pathways	ICU-like standard care Loss of mass and dysfunction Altered protein balance Mechanical ventilation and immobilisation Long term model	No critical illness Labour intense	[22,172]
**Burn injury**						
Mice	Inflammation	Fluid resuscitation and analgesic	Muscle atrophy and dysfunction Mitochondrial dysfunction Increased catabolism Decreased protein signalling pathways	ICU-like standard care Muscle atrophy and dysfunction Inflammation Altered protein balance	No mechanical ventilation or immobilisation	[173,174]
Rats	Inflammation	Fluid resuscitation and analgesic	Muscle atrophy and dysfunction Increased catabolism Reduced protein synthesis pathways	ICU-like standard care Muscle atrophy and dysfunction Inflammatory response Altered protein balance	No mechanical ventilation or immobilisation	[175–177]
**Immobilisation (limb casting and hindlimb suspension**)						
Mice	Immobilisation	N/A	Muscle atrophy Increased catabolism Reduced protein synthesis	Muscle atrophy Altered protein balance Anabolic resistance Immobilisation	No critical illness No mechanical ventilation	[178–180]
Rats	Immobilisation	N/A	Muscle atrophy Increased catabolism Reduced protein synthesis	Muscle atrophy Altered protein balance Anabolic resistance Immobilisation	No critical illness No mechanical ventilation	[181,182]
**Patient serum**						
C2C12 cells	Critical illness	N/A	Atrophy with loss of myosin Increased catabolism	Isolation of muscle only effect Atrophy and increased catabolism	Lack of systemic interaction	[183]
Human primary cells	Critical illness	N/A	Aberrant autophagy	Differential autophagy response	Lack of systemic interaction	[184]

#### a) Lipopolysaccharide

LPS, a form of endotoxin, is found in the cell wall of all gram-negative bacteria [185] and released from the cell after death [186]. These endotoxins can be isolated and purified [187] for use in eliciting a sterile inflammatory response in model organisms. LPS binds to LPS-binding protein (LBP), which activates the CD14 receptor on the cell membrane of immune cells [188]. The interaction of CD14 with MD2 facilitates signalling through TLR4 to elicit a downstream inflammatory response [189]. LPS is not a model of sepsis *per se*, since it does not produce a prolonged, sustained increase in circulating cytokines [126]. To circumvent this shortcoming, some studies provide a continual infusion of LPS to host organisms, although this scenario is not representative of how septic patients are normally exposed to a pathogen [126]. LPS is used to model muscle inflammation and wasting associated with a systemic inflammatory insult.

LPS exposure significantly reduces muscle mass in mice [150,153,190], rats [157–159] and guinea pigs [162], with the initiation of wasting occurring as early as 24 hours later with higher doses (5 mg/kg) [191]. Loss of muscle mass and decreased forelimb grip strength have been reported in mice ten days after LPS exposure [150]. Variation in the wasting phenotype is caused by variability in the dose, with concentrations ranging from 1 to 50 mg/kg. LPS administration (*i.p*.) also results in a loss of muscle mass in the rat diaphragm 48 hours after exposure, which preceded the reduction in specific force of isolated diaphragm muscle strips *ex vivo* [157]. Studies using LPS in pigs have incorporated ICU-like standard care to maintain the haemodynamic system and allow for examination of additional risk factors for muscle wasting, including mechanical ventilation and immobilisation [163–165]. Skeletal muscle dysfunction was observed in pigs five days after LPS exposure, with single fibres isolated from the biceps femoris muscle having reduced specific force across all fibre types [166]. Large animal models are commonly used to examine how inflammation affects nutritional feeding strategies and how anabolic resistance may limit the efficacy of nutritional interventions [151,192,193].

The use of endotoxin models as sepsis surrogates is common, but these do not recapitulate the inflammatory state of true sepsis. Rather, they serve to investigate the initiation of a muscle wasting phenotype associated with acute systemic inflammation. LPS remains a useful and reproducible model for understanding mechanisms associated with anabolic resistance in response to inflammation and the associated muscle wasting.

#### b) Zymosan

The zymosan model of peritonitis was originally developed to demonstrate that a sterile inflammatory response could mimic signs of sepsis and cause multiple organ failure [194]. Extracted from the cell wall of the fungus *Saccharomyces cerevisiae,* zymosan activates a systemic inflammatory response through various mechanisms, including TLR-2 [195,196] and the host’s complement system [196,197]. Unlike other inflammatory models, the suspension of zymosan in liquid paraffin allows for a sustained inflammatory response, which more closely resembles the phenotype of multi-organ dysfunction in critically ill patients [167,168,196].

The zymosan model has been used to study mechanisms of skeletal muscle wasting and weakness with critical illness, particularly with respect to the recovery of muscle mass and function once the illness has resolved [167,168,198–206]. Recovery of muscle mass and function was evident at 12–14 days post zymosan administration in rats. The peak reduction in hindlimb force *in situ* occurred on day 2 [167] and at day 6 [199,204], with the loss of functional capacity consistent with the muscle atrophy. Functional recovery, based on treadmill running duration, occurred 14 days after zymosan administration [167].

Unlike terminal models of critical illness, or long-term models of dysfunction such as the CS, the zymosan model facilitates investigation of mechanisms responsible for the recovery of muscle mass and function. The translational significance of the zymosan model would benefit from further studies investigating how risk factors/variables such as age and sex affect skeletal muscle structure, function and metabolism during critical illness, particularly whether aged mice can similarly recover from such an insult.

### Hypercatabolic models

#### a) Burn injury

One of the hallmarks of many of the models presented herein is acute hypothermia at the onset of sepsis, with a concomitant reduction in oxygen consumption and metabolic rate [167,207–209]. However, in clinical situations, such as burn injury, there is a hypercatabolic response which also leads to significant muscle wasting and weakness. Burn injuries are a common presentation to the ICU, and patients often experience a loss of muscle mass and function [210]. While burn injury is characterised by a systemic inflammatory response like that experienced by most critically ill patients, the type of burn injury, i.e., thermal, electrical, or chemical, can cause differential effects on the skeletal muscles of patients [210]. Burn models in mice [173,174], rats [175–177] and rabbits [211] have incorporated fluid resuscitation and analgesics in their interrogation of skeletal muscle wasting. Muscle atrophy and dysfunction due to increased catabolism and decreased protein synthesis have been reported in these model systems. Burn injury provides an alternative route of critical illness induction, but further investigations of the differential muscle responses based on the type of burn injury could provide mechanistic insight for optimising therapeutic development.

### Combination and risk factor models

Some models of critical illness are not initiated directly by inflammation and are used to investigate other risk factors associated with the development of muscle wasting and weakness in the ICU (Figure 1, Table 1). Patients admitted to the ICU may be affected by other compounding factors that exacerbate muscle wasting and weakness. Incorporating these risk factors into pre-clinical models can provide insight into the mechanisms underlying muscle dysfunction and are important in the development of effective therapeutics.

#### a) Corticosteroids with denervation

Patients in the ICU are often sedated and immobilised and so to examine the effect of this ‘mechanical silencing’ and the local and systemic environments that promote muscle wasting, sciatic nerve denervation in rats has been combined with corticosteroid treatment for a two-pronged insult that results in muscle wasting and weakness like in critically ill patients [169]. In this model, hallmarks of wasting and weakness include muscle atrophy [169,170] after seven days and a preferential loss of myosin, findings similar to those observed in patients with critical illness myopathy, a subset of ICU-AW [8]. In that study, muscle dysfunction was based on the reduction in specific force of rat plantar flexor muscles *in situ* [169]. Electrophysiological abnormalities have also been reported in this model, with muscle fibres exhibiting a hyperpolarising shift in the voltage dependence of fast inactivation of Na^+^ channels responsible for their inexcitability [171].

Although one of the few models to mimic the reduction of the myosin/actin ratio like that in critically ill patients, this model does not involve a systemic inflammatory response like that typically presented by patients in the ICU. The ‘mechanical silencing’ in this model also differs from that in patients where the nerve is primarily intact and trophic signalling from the motor endplate is not compromised. These differences highlight potential limitations in translating findings from the animal model to the clinic.

#### b) Mechanical ventilation and neuromuscular blockade

Interventions to treat critically ill patients in the ICU can also contribute to muscle wasting and weakness. These include mechanical ventilation, immobilisation and use of neuromuscular blocking agents [8]. Insights into the influence of these risk factors on skeletal muscle have come from studies utilising a model where rats are sedated, mechanically ventilated, and administered neuromuscular blocking agents [22,172]. In this environment, rats exhibit atrophy, dysfunction and altered protein metabolism in the hindlimb muscles [172] and diaphragm [22]. Hindlimb muscle dysfunction is evident at the single fibre level after 5 days, with reduced specific force of soleus muscle fibres [212], attributed to impaired Ca^2+^ signalling and reduced sarcoplasmic reticulum (SR) Ca^2+^ release after eight to ten days [213]. The hindlimb muscle wasting associated with a reduced myosin/actin ratio was similar to that in patients with critical illness myopathy [172]. However, the myosin/actin ratio was unchanged in the diaphragm muscles of these rats, indicating a possible different mechanism of wasting compared with that in hindlimb muscles [22]. Muscle dysfunction was evident even within six hours after onset of mechanical ventilation, with the diaphragm pathology progressively worsening, and functional impairments preceding muscle fibre atrophy.

While this model provides important insights into mechanical ventilation dysfunction, ICU-AW is induced in the absence of systemic inflammatory mediators and differs from the critical illness experienced by patients. Furthermore, continual heavy sedation and use of neuromuscular blocking agents throughout mechanical ventilation only occurs in a limited number of ICU patients, with many requiring only light sedation without neuromuscular blockade, limiting some aspects of its clinical relevance.

#### c) Disuse

Disuse is one of the main risk factors for the development of acquired myopathies in the ICU. In rodents, models such as hindlimb suspension (HLS) and limb casting are often used as surrogates of the ICU environment (Table 1). These include skeletal muscle atrophy [178,181,214] after 7 [178] and 14 [214] days of disuse, and reduced puromycin incorporation after 3 days of disuse [179], indicating impaired protein synthesis. However, disuse models in isolation do not replicate the systemic inflammation in critically ill patients and do not account for long-term dysfunction, even after muscle loading and normal nutrition have been restored.

HLS protocols have been combined with critical illness models, such as CLP-induced sepsis [59,145], to try and provide greater insight into the mechanisms underlying ICU-AW. The combination of CLP followed by seven days of HLS, four days after sepsis induction, induces more severe muscle atrophy [145], and remodelling of the entire muscle proteome [215] compared with sepsis alone. HLS was associated with greater inflammatory response [59] and exacerbated dysfunction of the soleus muscle compared with sham and CLP-only groups, with impairments in *ex vivo* measures of maximum absolute force and specific force [59,145]. However, HLS models are more closely representative of microgravity environments compared with the disuse observed in the ICU [216].

Limb casting protocols, which involve complete immobilisation of the affected limb, more closely resemble the disuse observed in the ICU. Limb casting in combination with CLP-induced sepsis exacerbated the muscle atrophic response and reductions in hindlimb grip strength in mice compared with mice with sepsis alone [217]. However, the effects of limb casting models are only isolated to the limbs. In the ICU, patients are often mechanically ventilated and experience complete whole-body mechanical silencing of the skeletal muscles. Limb casting models facilitate investigation of the effect of critical illness in an ICU-like environment on diaphragm pathology not induced by mechanical ventilation. A combination of models may provide greater insight into the muscle impairments experienced by patients in the ICU and is important for the development of effective interventions.

### *In vitro* models of muscle wasting and weakness associated with critical illness

Animal models are not the only useful system for interrogating mechanisms underlying muscle wasting and weakness associated with critical illness. Muscle cell culture systems facilitate investigation of how circulating factors affect skeletal muscle cells, helping to identify causes of muscle atrophy and weakness. For example, endotoxins such as LPS provide an effective and reproducible way of inducing inflammatory responses *in vitro*. In mono-culture systems of a single cell type, such as the immortalised murine muscle C2C12 cell line, administration of LPS at various stages of the cell cycle induces an inflammatory response from the muscle cells. Compared with *in vivo* models where LPS elicits an immune response that activates a systemic inflammatory response which can affect the muscle, the culture experiments permit evaluation of the muscle cell response directly and in isolation.

LPS exposure reduces myotube diameter of C2C12 muscle cells after 24 [151] and 48 hours [150,153]. *In vitro* experiments permit analysis of these inflammatory agents at different stages of myogenesis, including proliferation and differentiation. C2C12 myoblasts exposed to LPS had an impaired differentiation capacity [218], with implications for explaining compromised muscle regeneration after a bout of sepsis [219]. Mature C2C12 myotubes treated with recombinant IL-6 exhibited a wasting phenotype like that with LPS, as early as 24 hours [100]. However, the systemic inflammation associated with critical illness does not simply involve elevation of a single cytokine. Therefore, supplementing cells with serum isolated from critically ill patients allows for a more patient-relevant assessment of how circulating factors can affect skeletal muscles directly [183]. Similarly, co-culture systems investigating LPS activation of macrophages cultured with C2C12 muscle cells [154] may better replicate a more physiological environment. Rather than utilising immortalised cell lines, a system utilising human primary myoblasts isolated from the vastus lateralis muscles of otherwise healthy patients revealed aberrant autophagic responses when incubated with the serum from critically ill patients [184]. Application of serum from critically ill patients on isolated cell systems *in vitro* may facilitate investigation of the circulating factors influencing human skeletal muscle, thus increasing the clinical translation and relevance of these studies.

While cell culture systems can be used to screen anti-inflammatory and anti-catabolic effects of different interventions, they are limited by not adequately modelling the organ-system interactions within a whole body, integrated physiological system. Three-dimensional (3-D) organoid culture systems derived from primary myoblasts are more like skeletal muscle than typical two-dimensional cell culture systems. Three-dimensional muscle organoid culture systems, or ‘micro muscles’, can be electrically stimulated to contract and forces measured [220]. Such 3-D systems can be utilised as a high-throughput method for drug discovery relevant to critical illness, with applications potentially using conditioned media from macrophages stimulated with LPS or incubated with serum derived from patients. Despite the usefulness of *in vitro* systems in facilitating investigation of muscle-specific effects, these systems are generally representative of immature skeletal muscle with limited expression of more mature myosin isoforms. Other factors associated with the development of ICU-AW, such as disuse, are also difficult to assess *in vitro*.

## Conclusions and perspectives

### Summary

Skeletal muscle wasting and weakness associated with critical illness impairs quality of life and is associated with prolonged dysfunction post-discharge from the ICU. Despite this burden of disease on patients, there are currently no effective therapies. Treatment options are limited to the modification of disuse, recognising that early mobilisation may not be possible for all patients due to the severity of their illness or because of staffing limitations in the clinic. Hence, there is an urgent need to develop effective pharmacotherapies and nutritional interventions that can be administered to all patients alongside the standard of care. Understanding the mechanisms responsible for critical illness, muscle wasting and weakness will provide greater insight into the development of these treatments. Conducting mechanistic studies in the clinic is challenging because of interpatient variability and an inability to obtain sufficient muscle tissue from biopsies to perform comprehensive analyses. The most common muscles analysed in the context of critical illness in pre-clinical animal studies include the tibialis anterior, soleus and diaphragm muscles, but comprehensive comparisons between different muscle groups are not common. In addition, the quadriceps, which is the most biopsied muscle in patients [11,22,23], is rarely investigated in pre-clinical studies using animal models. Assessments of different muscle groups are important because fibre composition differs between these muscles, which affects their functional characteristics. The tibialis anterior and quadriceps muscles have a largely force-producing function, whereas the soleus muscle has more of a postural function and its fibre composition makes it resistant to fatigue. The diaphragm is the muscle of respiration, and critically ill patients often experience diaphragm dysfunction requiring mechanical ventilation, which itself can lead to intrinsic muscle weakness. Examining different muscle groups is important for answering key questions regarding the mechanisms underlying ICU-AW, including (1) Are all muscle groups equally affected in critical illness settings or does muscle fibre composition confer protection for some muscle groups? (2) What is the role of the diaphragm in the overall physical dysfunction (in some patients) after discharge from the ICU?

***Are current models sufficient?*** Models of critical illness can provide insight into the mechanisms of wasting and weakness in ICU patients. Cell culture systems provide a platform for examining how inflammatory stimuli affect isolated muscle cells and facilitate evaluative screening of potential therapies to protect muscles from atrophy. However, cell culture systems are typically representative of immature skeletal muscle (expressing less mature myosin isoforms) and lacking the physiological integration of animal models. Animal models can enhance understanding of critical illness and ICU-AW as they allow for investigation of inter-organ signalling and integrated systems physiology. This is essential for robust interrogation of how inflammatory signalling disrupts the molecular pathways regulating skeletal muscle homeostasis. Some of these models have employed ICU-like standard care, including fluid resuscitation, antibiotics and analgesics, and in some cases have utilised mechanical ventilation and immobilisation/disuse.

Despite the development of animal models of ICU-AW across different species, most models do not completely mimic critical illness/ICU settings in human patients, and to date, no effective treatments for muscle wasting and weakness have advanced to the clinic. Most animal models are not representative of typical ICU patient populations and might account for limited admission categories, such as mechanical ventilation or sepsis, and often study only young, healthy animals rather than older animals that would more closely mimic the age of older patients in the ICU. Few animal models exhibit the muscle weakness experienced by many survivors of critical illness during their (often prolonged) recovery after hospital discharge.

### Recommended pre-clinical workflow

There is currently no standard animal model that best replicates the hallmarks of ICU-AW. Ideally, an animal model would include the following characteristics: (1) be representative of the age of a typical ICU population. For rodent models, this would require using older rats or mice between 18–24 months of age, recognising that the availability of older rodents is usually limited and often cost-prohibitive; (2) mimic a critical illness phenotype that leads to multi-organ dysfunction in the absence of disuse; (3) include a component of disuse given that inactivity and lack of mechanical loading contribute to muscle atrophy and weakness; (4) include sedation and mechanical ventilation where feasible, recognising that such experiments are technically very challenging and labour intensive; (6) have a time course that more closely represents the development of ICU-AW (i.e. for seven days or longer); and (7) include the capacity to recover from critical illness to facilitate investigations into long-term physical dysfunction after ICU discharge. Large animal models, such as pigs, have a similar immune system to that in humans and facilitate repeated blood sampling, which more closely mimics clinical evaluations in ICU settings. However, they are often prohibitively expensive and not used widely. Smaller animal models, such as rats and mice, allow for higher throughput analyses and mechanistic studies through genetic manipulation. Despite their obvious limitations, cell and animal models can provide important insights into the mechanisms underlying critical illness and ICU-AW and are critical for the development and evaluation of therapies for potential clinical translation.

## Abbreviations

ADL: activities of daily living
ARDS: acute respiratory distress syndrome
CASP: colon ascendens stent peritonitis
CLP: caecal ligation and puncture
CS: caecal slurry
CSA: cross-sectional area
3-D: three-dimensional
EDL: extensor digitorum longus
EMG: electromyography
EMS: electrical muscle stimulation
ICU: intensive care unit
ICU-AW: ICU-acquired weakness
LBP: lipopolysaccharide binding protein
LPS: lipopolysaccharide
NMBs: neuromuscular blockers
PAMP: pathogen associated molecular pattern
PICU: paediatric intensive care unit
SOFA: Sequential Organ Failure Assessment
TA: tibialis anterior
TLR: Toll-like receptor
i.p.: intraperitoneal
i.v.: intravenous
